# Current Treatment of Acute and Chronic Hepatitis E Virus Infection: Role of Antivirals

**DOI:** 10.5005/jp-journals-10018-1216

**Published:** 2017-05-05

**Authors:** Ananta Shrestha, Birendra P Gupta, Thupten K Lama

**Affiliations:** 1 Liver Foundation Nepal, Kathmandu, Nepal; 2 Central Department of Biotechnology, Tribhuvan University, Kathmandu, Nepal

**Keywords:** Antiviral drugs, Epidemiology, Hepatitis E, Therapy.

## Abstract

Hepatitis E virus (HEV) infection results in nearly 20 million new infections, resulting in 70,000 deaths globally each year. Previously thought as a disease limited to developing nations with poor sanitation and hygiene, it is increasingly recognized that even the most developed nations are not spared. A clear dichotomy in epidemiology of HEV is noted between developing and industrialized nations. The HEV genotypes 1 and 2 are common in Asia and Africa and are transmitted mainly by contaminated drinking water. Sporadic as well as large-scale epidemics of acute hepatitis have been noted with HEV genotype 1 infection in developing countries of Asia and Africa. On the contrary, HEV genotypes 3 and 4 are common in industrialized nations and unlike genotypes 1 and 2, they are transmitted by consumption of raw meat products, fruits, and blood transfusion. Large epidemics have not been reported with HEV genotypes 3 and 4 and manifestation is usually indolent, though severe acute hepatitis has been reported.

**How to cite this article:** Shrestha A, Gupta BP, Lama TK. Current Treatment of Acute and Chronic Hepatitis E Virus Infection: Role of Antivirals. Euroasian J Hepato-Gastroenterol 2017;7(1):73-77.

## MORBIDITIES ASSOCIATED WITH HEV INFECTION

Acute hepatitis E infection is usually a self-limiting illness lasting 1 to 3 months with spontaneous resolution. Apart from morbidity associated with the symptoms, the mortality associated with hepatitis E virus (HEV) in immunocompetent subjects is very low, 0.1 to 4%.^1^ Since HEV manifests as self-limiting illness with very low mortality in low-risk population, specific antiviral therapies if any are not warranted. However, in selected high-risk population like pregnant women, people with underlying chronic liver diseases, and other comorbid illness, the mortality may be high as much as 25% and specific treatment against the virus if proven beneficial will be highly useful.

Until a decade earlier, HEV was thought to cause acute infection with spontaneous clearance of virus. Kamar et al^2^ reported failure to clear HEV infection in subjects receiving posttransplant immunosuppression. Further, progressive liver damage leading to chronic liver disease, rapid evolution into cirrhosis, and decompensation was also reported.^3^ Subsequently, immunosuppression in background of chemotherapy, HIV infection, and various organ transplantation were identified as risk factor for chronic HEV infection. However, chronicity in HEV has been reported only from European countries and almost all the cases being due to genotype 3 infections. Developing countries of Asia and Africa where HEV genotype 1 and 2 dominates, chronicity has not been reported. The maximum duration of viremia and fecal shedding in genotype 1 HEV infection was found to be 120 days and 30 days respectively among healthy subjects.^4,5^ Studies in North India, where HEV is endemic, failed to show persistence of HEV infection among transplant recipients.^6^ However, a single case report from New Delhi claims chronic infection due to HEV genotype 1 leading to cirrhosis in a child with acute leukemia that received chemotherapy.^7^

Specific antiviral therapy if available and efficacious is highly desired in certain groups of patients where risks of complications, mortality, or chronicity are high (Table 1).

**Table 1:** **Table 1:** HEV-associated conditions with adverse outcomes

• Acute liver failure	
• Acute on chronic liver failure	
• Severe acute hepatitis (non-ALF)	
• Acute HEV in pregnancy	
• Chronic HEV infection in immunosuppressed subjects	

## SPECIFIC ANTIVIRAL THERAPIES FOR HEV IN SPECIAL SITUATIONS

Ribavirin and interferon *a* has been used to treat cases of HEV infection. Most of these cases where antivirals have been used are in the setting of chronic HEV infection in immunosuppressed host. There are anecdotal reports of use of ribavirin in the setting of severe acute HEV infection with worsening liver function and acute on chronic liver failure (ACLF). *In vitro* experiments have demonstrated activity of sofosbuvir against HEV replication, but there have been no reports of its use in humans.

### Hepatitis E virus-induced Acute on Chronic Liver Failure

In Asia, HEV infection is an important cause of ACLF. Nearly 21% of cases of ACLF in Asian countries are accounted due to HEV infection, with one-third of them dying within 28 days.^8^ Acute on chronic liver failure is a distinct entity with well-understood natural history. Early treatment with antiviral in HBV flare-induced ACLF has been established. Following the same paradigm, there may be role of antivirals in improving outcomes of HEV-related ACLF. However, the data on efficacy and safety of Ribavirin in HEV-related ACLF are lacking. There have been a few anecdotal reports from both East and the West, but in the form of small case series (Table 2).^9-11^ Based on unreported experiences of experts gathered through personal communications in this meeting (Miyakawa Memorial Research Foundation, Tokyo, Japan 2016), it seems that ribavirin may improve outcomes in at least some of these patients if not all. Though the outcomes in these series seem appealing, properly designed, well-controlled trials are needed before anything can be commented on their utility.

### Hepatitis E virus-related Acute Liver Failure and HEV in Pregnancy

In South-East Asia, HEV infection is perhaps the commonest cause of acute liver failure (ALF). The relation between pregnancy and adverse fetomaternal outcome is well established, but its actual cause and pathogenesis still remains an enigma. Supportive intensive care and early transplant in selected candidates has been the mainstay of treatment over the last decade. There have been no reports of use of ribavirin in the setting of ALF and pregnancy.

Once ALF sets in, the natural course of disease is "explosive." The median time between presentation and death, transplant or recovery is 4.9 to 5.4 days.^12^ Given such nature of condition, it is very unlikely that use of any antivirals even early in course of disease would improve outcome. Further, ribavirin is contraindicated in pregnancy, limiting its use in this particular setting.

**Table 2:** **Table 2:** Outcome of ribavirin in HEV-induced ACLF

*Authors*		*HEV genotype*		*Number of cases*		*Outcome*	
Peron et al		3		2		2/2 cases recovered	
Goyal et al^9^		1		4		4/4 cases recovered	
Peron et al		3		3		2/3 cases died	

### Hepatitis E virus in Severe Acute Hepatitis (Non-ALF)

Acute hepatitis due to HEV has several clinical forms. Some of the patients develop protracted illness with worsening liver function over weeks. Unlike fulminant liver failure associated with HEV, sub-ALF and prolonged cholestasis is also seen. Some groups have used antivirals in such setting and found encouraging results. Ribavirin has been used in such cases of severe acute hepatitis E with worsening liver function with encouraging results. A multicenter retrospective study reported 21 cases of acute hepatitis due to HEV genotype 3 being treated with ribavirin.^12^ Nine of them had severe acute hepatitis and ribavirin was used till HEV ribonucleic acid (RNA) was negative. All the patients cleared the virus and ribavirin was well tolerated. Similarly among 13 patients of severe acute HEV who were treated in Bangladesh, ribavirin was reported to be safe and effective in improving liver function.^13^ However, the numbers of patients in these studies are small and they lack proper design and do not provide sufficient evidences to treat patients with acute hepatitis E patients.

### Chronic Hepatitis E virus Infection Related with Immunosuppression

It is now established that HEV genotype 3 can lead to chronic infection in transplant recipients and immuno-suppressed subjects. Worsening of liver function, liver cirrhosis, and decompensation due to chronic HEV infection can occur as early as 2 years of its onset.

After initial infection in immunosuppressed subjects, HEV may spontaneously clear in nearly two-thirds of the cases within 3 months.^3^ Persistence of HEV RNA beyond 3 months is required to define chronicity. While use of tacrolimus, low levels of CD4+ and CD8+, shorter duration since transplant, and recent acute rejection were associated with chronicity, mycophenolate mofetil was found to favor spontaneous clearance.^3,14,15^ Thus, it becomes logical to reduce the dosage of tacrolimus while building up the dose of mycophenolate mofetil during the first 3 months of detection of HEV infection to facilitate spontaneous clearance of the virus. After observing for the duration of 3 months, if HEV RNA still persists, then antiviral therapy may be started.

**Graphs 1A to C:**
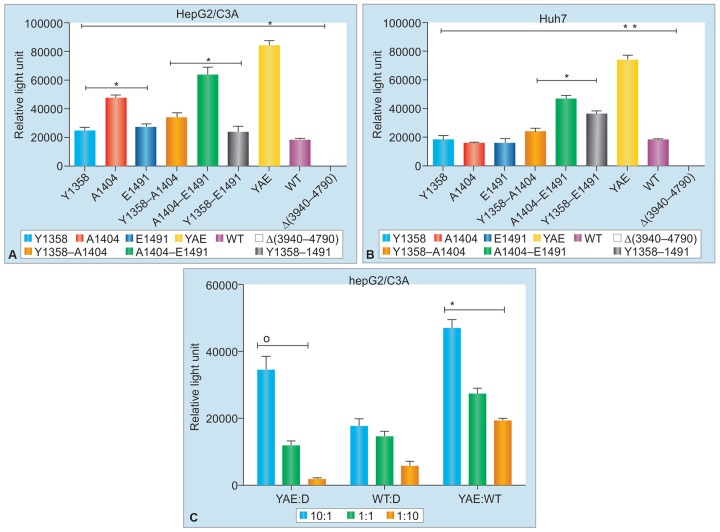
The HEV isolates from epidemic of Nepal with mutations in RNA-dependent RNA polymerase in HepG2/C3A and Huh7 cell lines. Presence of all three mutations, viz. Y, A, E, shows augmented replication fitness

**Graphs 2A to C:**
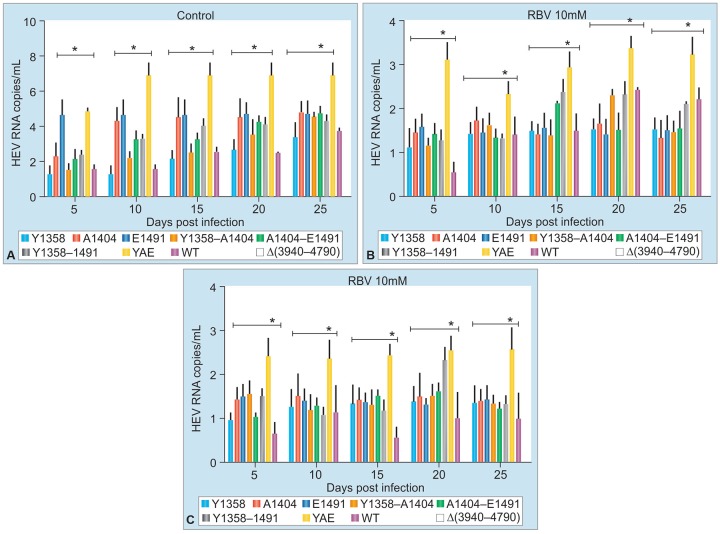
The HEV isolates containing all three mutations (viz. Y, A, E) conferring replication fitness were less sensitive to suppression *in vitro* by ribavirin

Kamar et al^16^ first published a series of three cases of chronic HEV infection in liver transplant recipients who were treated with interferon-alpha. Two of them attained sustained virologic response (SVR) while one relapsed. However, there was need for another agents, as interferon could not be used in solid organ transplants other than liver for the risk of rejection. The same group then treated six cases of chronic HEV infection with ribavirin for 3 months and SVR was achieved in four cases.^17^ Subsequently, there have been several reports of successful use of ribavirin in chronic HEV infection, with overall SVR of 79%.^18^ Relapse was noted in nearly one-fifth of the cases treated with ribavirin. Protracted fecal shedding during ribavirin therapy was associated with higher relapse rate. Further, selection of HEV variants with mutation in RNA-dependent RNA polymerase (RdRp) gene at G1638R was associated with increased replication fitness and higher relapse rates.^19^ Similar observations were made by our group in HEV isolates from an epidemic in eastern city of Nepal. Three different mutations were noted in RdRp gene of these isolates that provided increased replication fitness (Graphs 1A to C) and decreased sensitivity to ribavirin in cell culture models (Graphs 2A to C).

Based on currently available evidences, 3 months of ribavirin is advisable as initial treatment of chronic HEV infection.^20^ Persistence of HEV in stool or serum after 3 months of ribavirin therapy should urge extension of duration of therapy by 3 more months. In case of relapse after initial therapy, another course of 6 months of riba-virin therapy is advised. Pegylated interferon therapy for 3 months may be considered for liver transplant recipients with detectable HEV RNA in serum and stool after 6 months of ribavirin therapy.

## CONCLUSION

In both developing and developed nations, HEV is an important cause of liver disease. Previously considered as a self-limiting disease not requiring specific therapy, now it has been increasing need for specific therapy in various spectrum of diseases caused by HEV. While antiviral therapy is well established for chronic HEV infection, there are inadequate evidences for antiviral use in various spectrum of acute hepatitis including ACLF and severe acute hepatitis E. There is a definite need for a well-designed study to assess safety and efficacy of ribavirin in severe acute hepatitis and ACLF induced by HEV.
